# Long response duration to pembrolizumab in metastatic, castration-resistant prostate cancer with microsatellite instability-high and neuroendocrine differentiation: A case report

**DOI:** 10.3389/fonc.2022.912490

**Published:** 2022-09-16

**Authors:** Tsukasa Yoshida, Hiroshi Yaegashi, Ren Toriumi, Suguru Kadomoto, Hiroaki Iwamoto, Kouji Izumi, Yoshifumi Kadono, Hiroko Ikeda, Atsushi Mizokami

**Affiliations:** ^1^ Department of Integrative Cancer Therapy and Urology, Graduate School of Medical Science, Kanazawa University, Kanazawa, Japan; ^2^ Department of Pathology, Kanazawa University Hospital, Kanazawa, Japan

**Keywords:** metastatic castration-resistant prostate cancer (mCRPC), neuroendocrine differentiation (NED), microsatellite instability-high (MSI-high), immune checkpoint inhibitor (ICI), pembrolizumab

## Abstract

**Background:**

The detection of microsatellite instability in urologic cancers is rare, especially in metastatic, castration-resistant prostate cancer with neuroendocrine differentiation.

**Case presentation:**

This is a case of a 66-year-old Asian male patient with prostate adenocarcinoma who had metastases at initial presentation. Despite combined androgen deprivation therapy, his prostate-specific antigen (PSA) progressively increased, and prostate re-biopsy revealed small cell carcinoma. He was treated with platinum-based systemic chemotherapy, and his tumor markers, including PSA, remained negative; however, his local symptoms worsened. Subsequently, microsatellite instability-high was detected, and pembrolizumab was administered resulting in complete remission with the resolution of symptoms and continued therapeutic effect for more than 14 months.

**Conclusion:**

Microsatellite instability testing should be considered, despite its low detection rate, because the response to pembrolizumab in metastatic, castration-resistant prostate cancer with detectable microsatellite instability is associated with a prolonged duration of response.

## Introduction

Small cell/neuroendocrine (NE) differentiation in prostate cancer can appear *de novo* in untreated patients but is relatively rare (<2%) (1). More commonly, NE differentiation occurs in castration-resistant patients after androgen deprivation therapy (ADT) (2). Recent studies have pointed to a model of divergent clonal evolution from castration-resistant prostate cancer (CRPC)-adenocarcinoma to CRPC with NE differentiation (CRPC-NE) with adaptation from an androgen receptor (AR)-driven state to an AR-independent state (3).

The limited therapeutic options for treating NE prostate cancer include cisplatin, carboplatin with etoposide (4), or docetaxel with a marginal median survival of 7–15 months (5–7).

Pembrolizumab, an anti-programmed cell death (PD)-1 monoclonal antibody, is known to exhibit antitumor activity in advanced non-small cell lung cancer (8), gastric cancer (9), urothelial carcinoma (10), and malignant melanoma (11). It has recently been considered to be an effective treatment for patients with microsatellite instability (MSI)-high and mismatch repair-deficient (dMMR) cancer (12). The frequency of MSI-high or dMMR in prostate cancer is not great ranging from 3% to 22% (13–18).

This report describes, to the best of our knowledge, the first case of metastatic CRPC-NE with MSI-high that responded significantly to pembrolizumab and produced a long duration of response.

## Case presentation

**Figure 1:**
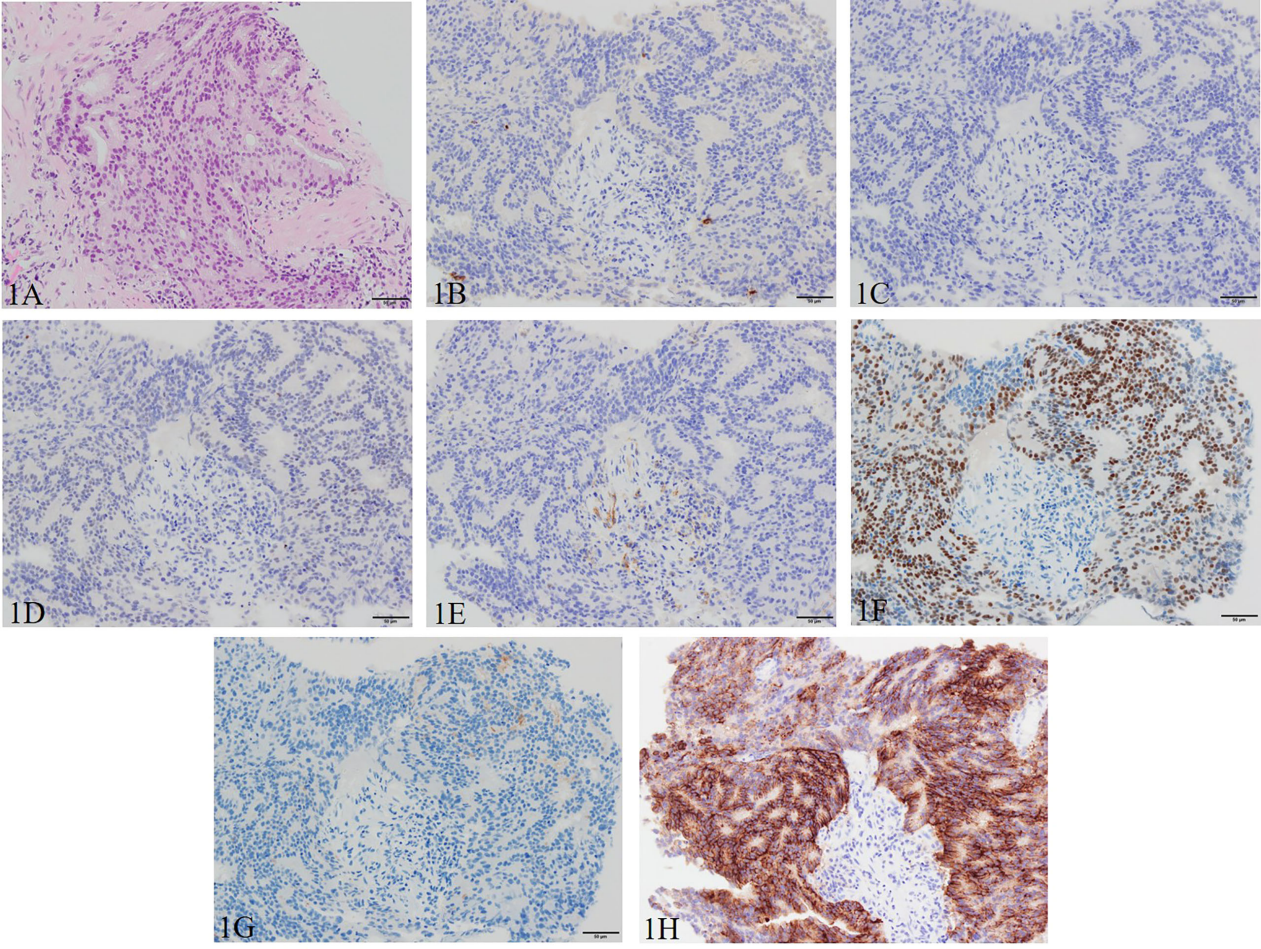
Pathological manifestations of initial prostate biopsy. Hematoxylin and eosin staining (magnification, ×200): **(A)** Gleason pattern 4 adenocarcinoma with nuclei unevenly distributed, cribriform, and growing in the form of fused glands is identified. Immunohistochemistry (magnification, ×200): **(B)** chromogranin A, **(C)** synaptophysin, **(D)** INSM1, and **(E)** SSTR2 are negative, and **(F)** AR, **(G)** PSA (focal), and **(H)** PD-L1 (focal) are positive. AR, androgen receptor; INSM1, insulinoma-associated protein 1; PD-L1, programmed death ligand-1; PSA, prostate-specific antigen; SSTR2, somatostatin receptor 2.

A 66-year-old Asian male patient first presented to another hospital with complaints of a sense of residual urine and pollakiuria in March 2019. There was no family history of malignancy to the fourth degree of consanguinity, and the patient himself had no history of other malignancies. The prostate-specific antigen (PSA) was 50.2 ng/ml (<4.0 ng/ml), resulting in a suspicion of prostate cancer. Transrectal prostate needle biopsy was performed in April 2019 resulting in a Gleason score of 4 + 4 adenocarcinoma (**Figure 1**). The tumor proportion score (TPS) of PD-L1 was approximately 50% using the clone 22C3 pharmDx kit (Agilent Technologies, Inc., Santa Clara, CA, USA) (**Figure 1**).

**Figure 2:**
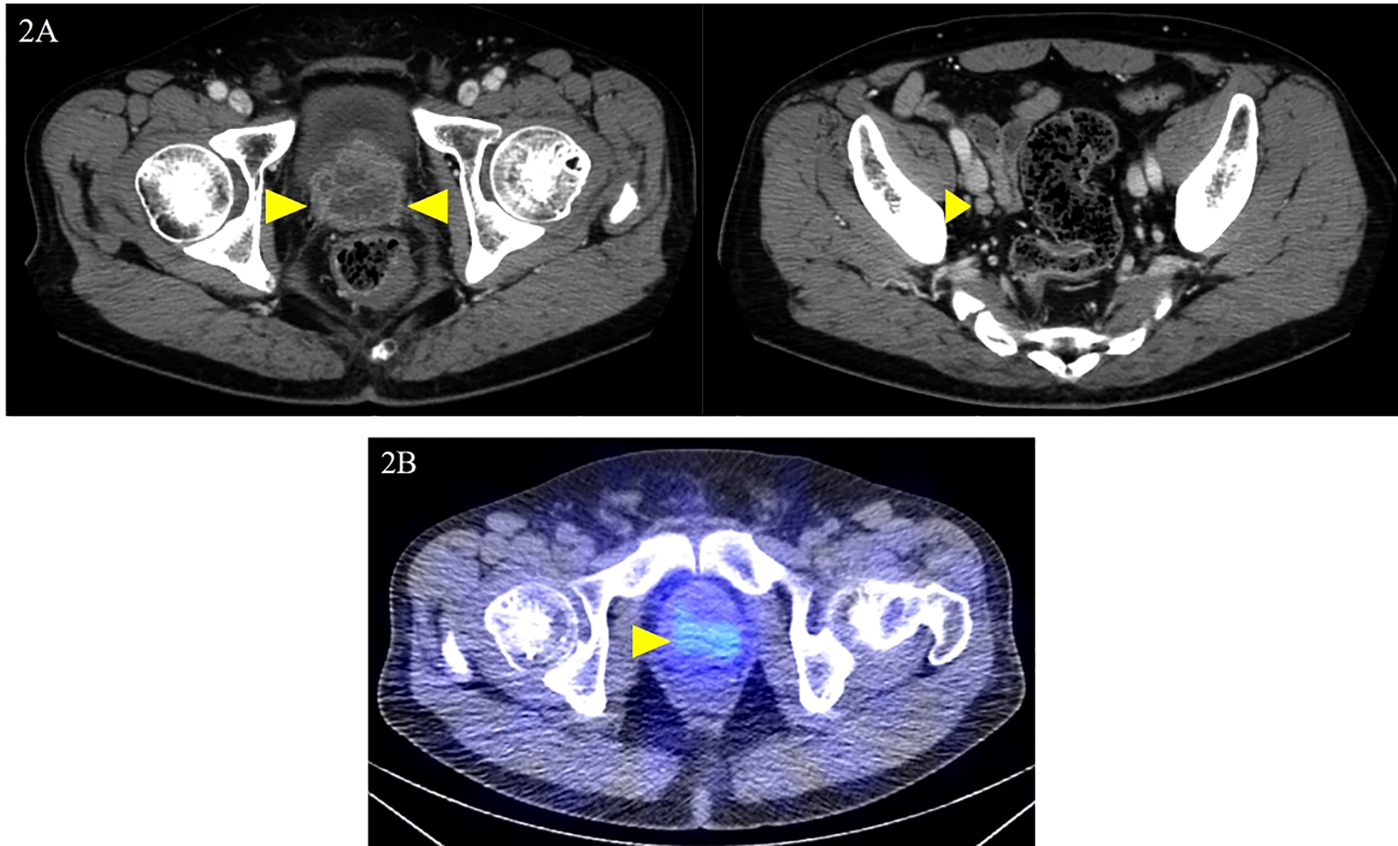
Local prostate lesion and involved lymph nodes on contrast-enhanced computed tomography and somatostatin receptor scintigraphy. Contrast-enhanced computed tomography examination at the first visit to the department: **(A)** dark staining throughout the prostate and enlarged right obturator lymph node was noted (yellow arrowheads); **(B)** somatostatin receptor scintigraphy shows mild accumulation in the local prostate lesion (yellow arrowhead).

**Figure 3:**
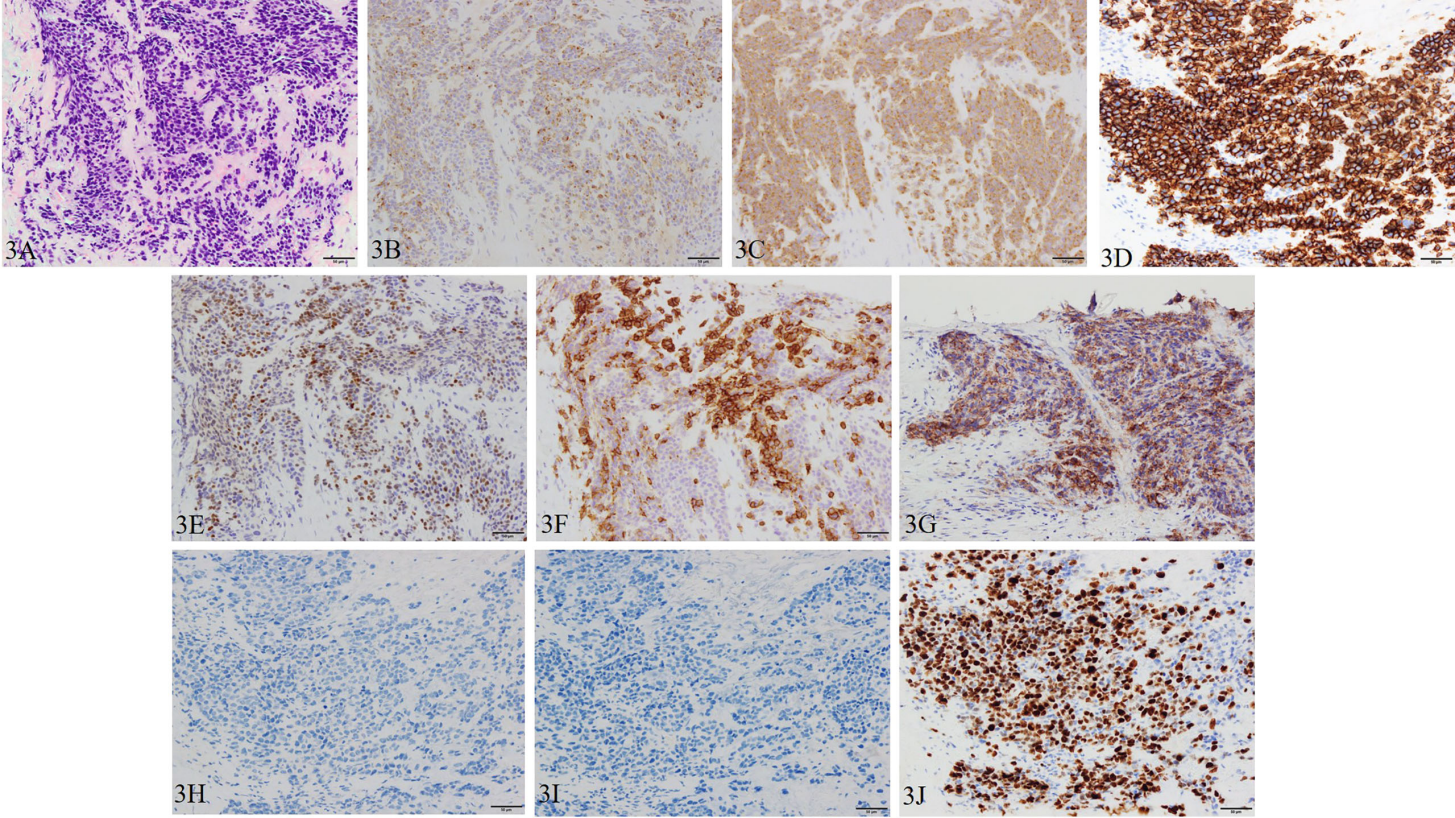
Pathological manifestations of prostate re-biopsy. Hematoxylin and eosin staining (magnification, ×200): **(A)** the tumor tissue is infiltrated with chromatin-rich, naked nucleated tumor cells with an increased nucleus/cytoplasm ratio of irregular nuclear shape. Immunohistochemistry (magnification, ×200): **(B)** chromogranin A, **(C)** synaptophysin, **(D)** CD56, **(E)** INSM1, **(F)** SSTR2, and **(G)** PD-L1 (focal) are positive, and **(H)** AR and **(I)** PSA are negative. **(J)** Ki-67 is also positive with a Ki-67 labeling index over 90%.

**Figure 4:**
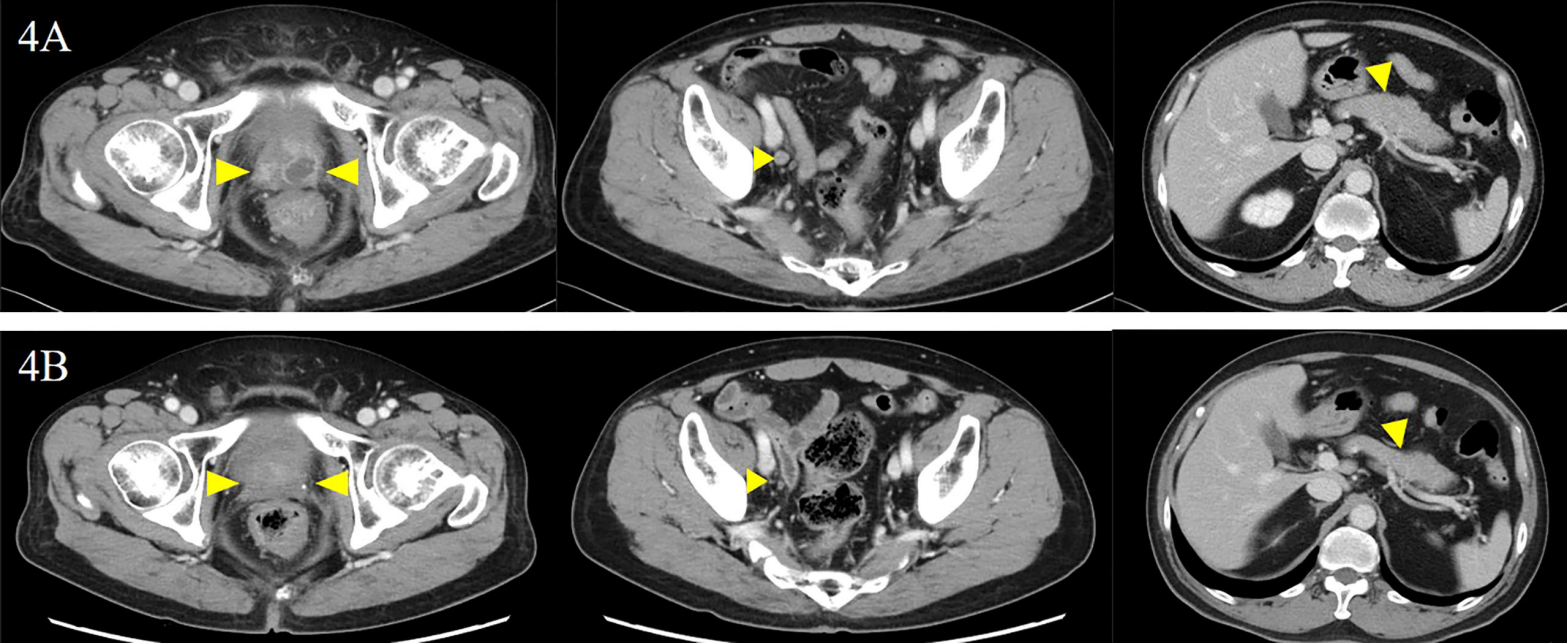
Local prostate lesion imaging including involved lymph nodes and pancreatic lesion before and after the administration of pembrolizumab. Contrast-enhanced computed tomography examination: **(A)** before the administration of pembrolizumab and **(B)** 5 months after the administration of pembrolizumab. There was complete remission of each lesion (yellow arrowheads).

The patient was diagnosed with cT3aN0M1c (PUL) prostate cancer, and combined androgen blockade (CAB) with bicalutamide and degarelix was administered. In August 2019, the PSA decreased to 1.151 ng/ml, but it rose again to 3.800 ng/ml in November 2019, and the patient was referred to our hospital for further treatment. Serum PSA, neuron-specific enolase (NSE), and progastrin-releasing peptide (proGRP) were present at high levels at the first visit to our department with values of 4.220 ng/ml, 24.3 ng/ml (<16.3 ng/ml), and 206 pg/ml (≤75 pg/ml), respectively. Serum testosterone was at a castration level of 0.05 ng/ml. Contrast-enhanced computed tomography (CECT) showed an irregular contrast effect over the entire prostate, and somatostatin receptor scintigraphy showed mild accumulation in the prostate (**Figure 2**). After changing from degarelix to leuprorelin acetate, a prostate re-biopsy was performed in December 2019, and small cell carcinoma was detected (**Figure 3**). The patient had metastatic CRPC-NE and was treated with etoposide plus cisplatin (EP) (etoposide, 100 mg/m^2^/day, days 1–3; cisplatin, 80 mg/m^2^/day, day 1, repeated every 3 weeks). After the first EP course, renal function declined, so cisplatin was replaced by carboplatin (CBDCA) with a total of two courses of etoposide plus CBDCA (etoposide, 80 mg/m^2^/day, days 1–3; CBDCA: AUC 5, day 1, repeated every 4 weeks). This was followed by irradiation of the prostate region (external beam radiation therapy, 70 Gy, 35 fractions). However, after completing irradiation, the patient complained of perineal pain and pain during urination, and CECT showed recurrent prostate staining, lymph node metastasis, and *de-novo* pancreatic metastasis. Two courses of single agent CPT-11 (100 mg/m^2^/day, days 1, 8, and 15, repeated every 5 weeks) were administered as salvage chemotherapy, and concurrent MSI was investigated. MSI status was investigated using an approved kit (MSI‐IVD kit, FALCO biosystems, Kyoto, Japan). The analysis of prostate re-biopsy specimens showed a high MSI status, and the patient was given pembrolizumab (200 mg/day, day 1, every 3 weeks). Following pembrolizumab administration, the local prostate, lymph node, and pancreatic metastases were all reduced, and complete remission was achieved with the resolution of symptoms (**Figure 4**). The patient is still undergoing treatment after more than 14 months of response without any immune-related adverse events. **Supplementary Figure 1** illustrates the transient changes in his PSA, NSE, and proGRP levels and the treatment course received by the patient.

## Discussion

MSI-high prostate cancer is rarely included as part of hereditary, non-polyposis colorectal cancer (HNPCC) also known as Lynch syndrome (19). However, the diagnosis of Lynch syndrome must meet Amsterdam criteria II, which at present requires at least three relatives with an HNPCC-related cancer, including cancers of the colon and rectum, endometrium, small bowel, ureter, or renal pelvis (20).

Although neuroendocrine-type prostatic adenocarcinoma with MSI-high in a patient diagnosed with Lynch syndrome has been reported previously (21), as described earlier, this patient does not meet Amsterdam criteria II, and he is, to the best of our knowledge, the first reported patient with non-hereditary CRPC-NE with MSI-high unrelated to Lynch syndrome worldwide.

In 2016, the World Health Organization reclassified prostatic NE tumors into five groups: standard adenocarcinoma with NE differentiation, adenocarcinoma with Paneth cell-like NE differentiation, carcinoid tumor, small cell NE carcinoma (SCNC), and large cell NE carcinoma (22). Following the classification of Hu et al., this case is consistent with what they designated SCNC (23). Additionally, *de-novo* SCNC is also rare, accounting for less than 1% of prostatic cancer cases (23).

However, in a multicenter prospective study, treatment-emergent SCNC was reported to account for 17% of the cases (24). Additionally, treatment-emergent neuroendocrine prostate cancer (T-NEPC) has been reported to occur primarily in advanced CRPC which is an alteration of normal prostate adenocarcinoma following ADT including CAB (25). Zhang et al. reported in a review of 94 cases of T-NEPC that 30.9% of AR and 47.9% of PSA were negative on immunohistochemical staining (26). Furthermore, T-NEPC has a median survival of 17.6 months. This is a significantly worse prognosis compared with normal CRPC patients (median survival of 23.6 months) with a reported median survival of 15.7 months for metastatic cases and 9.7 months for those with a small cell carcinoma component (26).

In this case, as shown in **Figure 1**, the pathology specimen of the initial prostate biopsy was weakly positive for PSA, strongly positive for AR, and negative for NEPC-related markers, but the pathology specimen of the re-biopsy turned negative for PSA and AR and positive for NEPC-related markers as shown in **Figure 3**. It is also a post-CAB state, and these facts strongly support that this case is T-NEPC. In addition, he had metastases in his lymph nodes and pancreas, and a small cell carcinoma component was detected on prostate re-biopsy which is consistent with a poor prognosis in T-NEPC. However, after confirming MSI-high, the patient survived more than 14 months, starting after pembrolizumab administration, without progression. It appears that even though it is infrequent, if MSI-high is present, a long-term survival benefit from pembrolizumab administration may be expected even in the poor prognosis group of T-NEPC. Furthermore, as noted previously, given that the detection rate of treatment-emergent SCNC is 17% (24), and the frequency of MSI-high in prostate cancer is rare, ranging from 3% to 22% (13–18), the frequency of cases with both MSI-high and T-NEPC is extremely rare. The patient in the present case had pancreatic metastasis. Although there are scattered reports of visceral metastases including bone, brain, liver, and lung in CRPC-NE (17, 26), we could not point to any pancreatic metastases as far as we could determine. There is a possibility that it is a feature of CRPC-NE with microsatellite instability-high, but this has not yet been confirmed and requires further investigation.

With regard to the patient’s perspective, Japanese health insurance covered all genetic testing, so the patient accepted all the tests. Because the Japanese insurance system permits only an outpatient setting when using a next-generation sequencer such as FoundationOne CDx^®^, we investigated only MSI with a kit in this case, which is an inpatient setting. Since this patient was symptomatic and also post-irradiation, MSI investigation was performed during CPT-11 administration. The timing of the outpatient visit was very difficult due to the patient’s ongoing symptoms and difficulty with chemotherapy withdrawal. Since next-generation sequencers (NGS) can detect many genetic mutations, the detection of mutations by NGS should be considered depending on the situation. However, if an outpatient visit is difficult during chemotherapy, MSI, which can be calculated separately from the NGS, may be considered first. Since the patient did not undergo a genomic test such as FoundationOne CDx^®^ in this case, the patient accepted the test using the MSI-IVD kit without any problems. However, if the disease progresses in the future, the FoundationOne CDx^®^ genomic test should be performed, and if the obtained results other than MSI-high indicate the hereditary nature of the disease, this fact should be explained to the patient after consultation with a physician specializing in genetic care.

It is necessary to discuss what treatment options will be available in the future if the disease progresses in the present case. First, a comprehensive cancer genome profiling using NGS will be done as it has not yet been performed. Depending on the results, there may be a therapeutic drug matching for the gene mutation. It has also been noted that Aurora kinase A inhibitor (27) and mammalian target of rapamycin inhibitor (28) may be effective against CRPC-NE although not in all cases. Next, immunohistochemical staining of the re-biopsy specimens showed expression of somatostatin receptor (SSTR) subtype 2. In addition, somatostatin receptor scintigraphy showed a mild accumulation of radionuclides in the prostate lesion. The usefulness of somatostatin receptor scintigraphy has been suggested not only in gastroenteropancreatic neuroendocrine tumors but also in CRPC-NE (29, 30). Furthermore, somatostatin receptor analogs such as octreotide have a high-binding affinity for SSTR subtypes 2 and 5 (31). These findings suggest that radionuclide therapy may be effective in this patient with SSTR subtype 2 positivity and somatostatin receptor scintigraphic accumulation.

The strength of the present case is that pembrolizumab, which was used in this case, is widely used in urologic malignancies and is relatively familiar with the management of immune-related adverse events. The limitation is that there are no other reports of durability of response in pembrolizumab in patients with CRPC-NE and MSI-high. Therefore, the optimal treatment for relapse after pembrolizumab treatment is still not well understood; however, the introduction of next-generation genome sequencers and positive immunostaining for SSTR subtype 2 in re-biopsy specimens should be used as a reference for treatment strategy.

## Conclusion

This report discusses a patient with a long-term response to pembrolizumab in CRPC-NE with MSI-high. It is noteworthy that while the patient had a small cell carcinoma component and metastasis to other organs and was in the poor prognosis group of T-NEPC, he still experienced long-term survival. If the presence of viable cells is suspected on radiological imaging, aggressive prostate re-biopsy should be performed. Furthermore, even if the results indicate a poor prognosis, MSI examination should be considered, even if its presence is infrequent.

## Data availability statement

The original contributions presented in the study are included in the article/**Supplementary Material**. Further inquiries can be directed to the corresponding author.

## Ethics statement

The studies involving human participants were reviewed and approved by the Ethics Committee of Kanazawa University. The patients/participants provided their written informed consent to participate in this study. Written informed consent was obtained from the individual(s) for the publication of any potentially identifiable images or data included in this article.

## Author contributions

TY and HIw treated the patient. TY and HY reviewed the literature and contributed to the preparation of the manuscript draft. TY and RT obtained the consent form from the patient. TY and HY drew the graph including the patient’s data and contributed to the preparation of the manuscript draft. HY and HIk interpreted the imaging and pathological findings. TY, HY, and AM were responsible for the revision of the manuscript and important intellectual content. All authors agree to be accountable for the content of the work. All authors contributed to the article and approved the submitted version.

## Funding

The open access publication fee will be carried by Kanazawa University.

## Acknowledgments

The authors would like to thank Enago (www.enago.jp) for English language editing.

## Conflict of interest

The authors declare that the research was conducted in the absence of any commercial or financial relationships that could be construed as a potential conflict of interest.

## Publisher’s note

All claims expressed in this article are solely those of the authors and do not necessarily represent those of their affiliated organizations, or those of the publisher, the editors and the reviewers. Any product that may be evaluated in this article, or claim that may be made by its manufacturer, is not guaranteed or endorsed by the publisher.

## Supplementary material

Supplementary Figure 1 Transitive graph of serum PSA, NSE, and ProGRP with clinical course. CE, carboplatin plus etoposide; CRPC-NE, castration-resistant prostate cancer with neuroendocrine differentiation; EP, etoposide plus cisplatin; NSE, neuron-specific enolase; proGRP, progastrin-releasing peptide; PSA, prostate-specific antigen; RT, external beam radiation therapy.
