# Supplementary figures and images for: Long response duration to pembrolizumab in metastatic, castration-resistant prostate cancer with microsatellite instability-high and neuroendocrine differentiation: A case report
Source: Front Oncol. 2022 Sep 16;12:912490. doi: 10.3389/fonc.2022.912490 (PMC9523122; doi:10.3389/fonc.2022.912490)

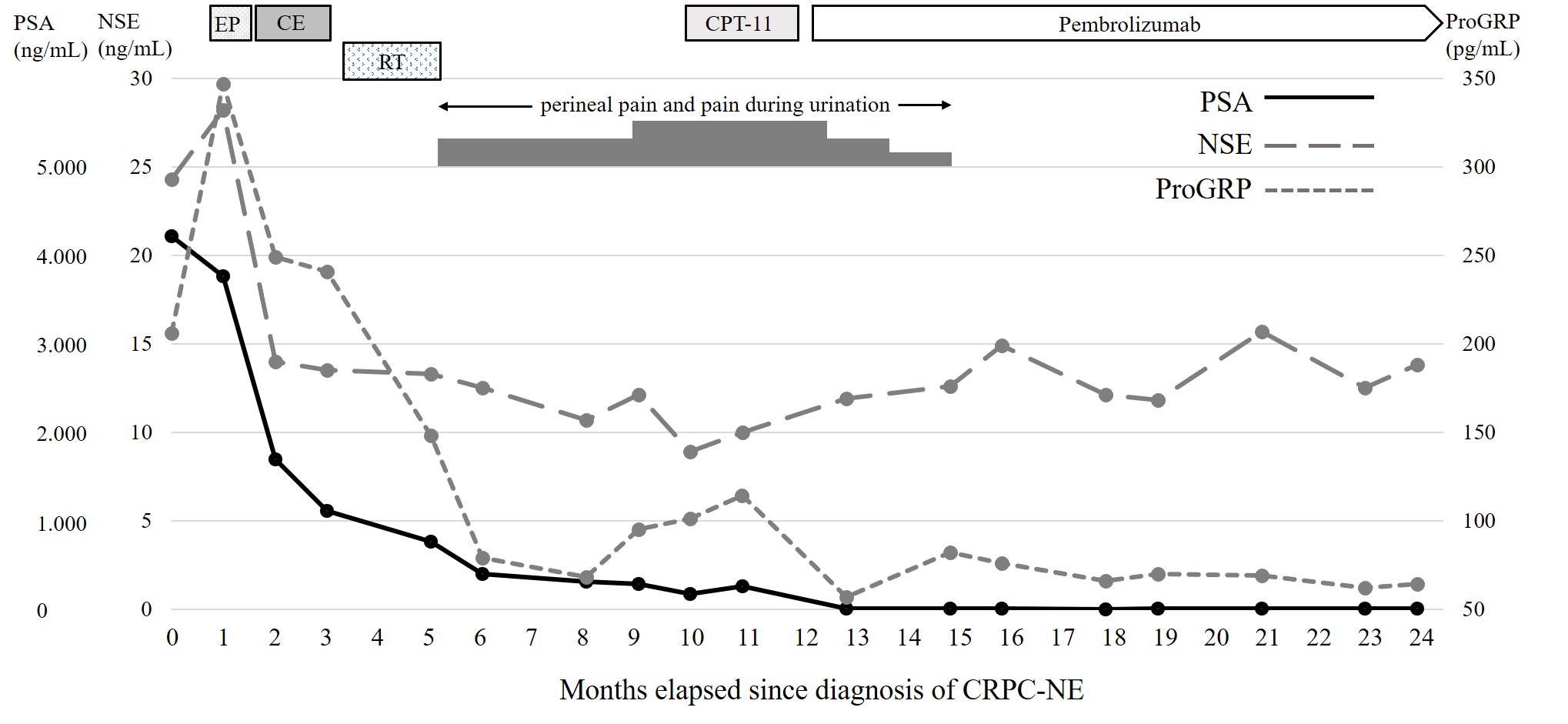

Supplement: Supplementary Figure 1 — Transitive graph of serum PSA, NSE, and ProGRP with clinical course. CE, carboplatin plus etoposide; CRPC-NE, castration-resistant prostate cancer with neuroendocrine differentiation; EP, etoposide plus cisplatin; NSE, neuron-specific enolase; proGRP, progastrin-releasing peptide; PSA, prostate-specific antigen; RT, external beam radiation therapy. [file Image_1.jpeg]
